# Enhancing kelp productivity in restoration and assisted adaptation interventions under ocean warming

**DOI:** 10.1038/s41598-025-22691-y

**Published:** 2025-11-05

**Authors:** Curtis Champion, Thomas Wernberg, Melinda A. Coleman

**Affiliations:** 1 NSW Department of Primary Industries and Regional Development, Coffs Harbour, NSW Australia; 2 National Marine Science Centre, Southern Cross University, Coffs Harbour, NSW Australia; 3 School of Biological Sciences, UWA Oceans Institute, University of Western Australia, Perth, WA Australia; 4 Norwegian Institute of Marine Research, Nye Flødevigveien 20, His, 4817 Norway

**Keywords:** Assisted adaptation, Blue carbon, Climate change, Intervention, Productivity, Restoration, Ecology, Ecology, Ocean sciences

## Abstract

Proactive climate interventions, such as restoration and assisted adaptation, are increasingly being used to secure the persistence of populations under climate change. Such strategies are being proposed globally for declining kelp forests, yet there remains a lack of consideration for potential impacts to other key traits that underpin their immense values. Here we assess the outcome of restoration and assisted adaptation strategies on kelp productivity – a key component of kelp forest blue carbon, habitat provision and ecosystem service values. We combine empirical field data, climate projections and knowledge of underlying genomics to compare kelp productivity across restoration and assisted adaptation scenarios that are, and are not, informed by productivity–temperature relationships within source populations. Using Western Australian kelp (*Ecklonia radiata*) forests, we show that the productivity of restored kelp using traditional strategies for selecting source material may decline by 16–22% under mid-century ocean warming. However, drawing on knowledge of productivity–temperature relationships to strategically select individuals could enhance the productivity of restored kelp by up to 9% even under mid-century ocean warming, while simultaneously boosting thermal resilience. Optimising trait interactions when undertaking climate interventions will be key to sustaining values associated with restored kelp forests in a warming ocean.

**Supplementary Information:**

The online version contains supplementary material available at 10.1038/s41598-025-22691-y.

## Introduction

Management and conservation of natural systems is increasingly moving towards proactive, interventionalist strategies to restore lost habitat and combat the impacts of climate change^1,2^. Given the natural ability of species to adapt to environmental change is increasingly being challenged^3^, these strategies not only seek to restore lost or degraded populations but are also now aiming to boost the ability of species to adapt to future climate change. In particular, strategies including assisted gene flow, assisted adaptation and assisted evolution are being proposed as pathways to boost resilience to climate stressors^1,2^. A key knowledge gap however, is whether there are trade-offs between selected and other key traits when climate interventions are undertaken. The aim of most assisted adaptation programs is to enhance thermal tolerance, yet it is often unknown if this occurs at the expense of other traits that contribute to species performance or ecosystem values.

Foundation species such as corals and kelps that underpin ecosystems are ubiquitously undergoing climate-mediated decline^4^ and are thus key targets for restoration and assisted adaptation programs given their disproportionate value in supporting biodiversity and ecosystem goods and services^5^. To date, assisted adaptation strategies for foundation species have focused on boosting resilience to climate change through identification of populations, individuals or genotypes with greater performance under warming^6,7^ or those with heritable genetic variation associated with higher ocean temperatures (e.g^8–10^. These individuals can then be propagated and used to restore or enhance threatened reefs to cope with warming^11^. While this may enhance the thermal tolerance of augmented populations, there can also be negative trade-offs between the thermal resilience of individuals or genotypes and other key traits such as growth or productivity^12,13^ (but see^14^). Moreover, how these trade-offs manifest under future climate scenarios is largely unknown. Understanding these trade-offs is therefore important for anticipating the outcomes of proactive climate interventions, but also for designing strategies that can potentially optimise outcomes for multiple traits. For example, kelp forest productivity underpins blue carbon sequestration and storage^15–17^ and sustains biodiversity and ecosystem functions in temperate marine systems^18,19^. Therefore, selecting individuals that are associated with relatively high productivity *and* thermal resilience for use in proactive climate interventions may have the potential to sustain (or even enhance) kelp forest values under climate change.

**Fig. 1:**
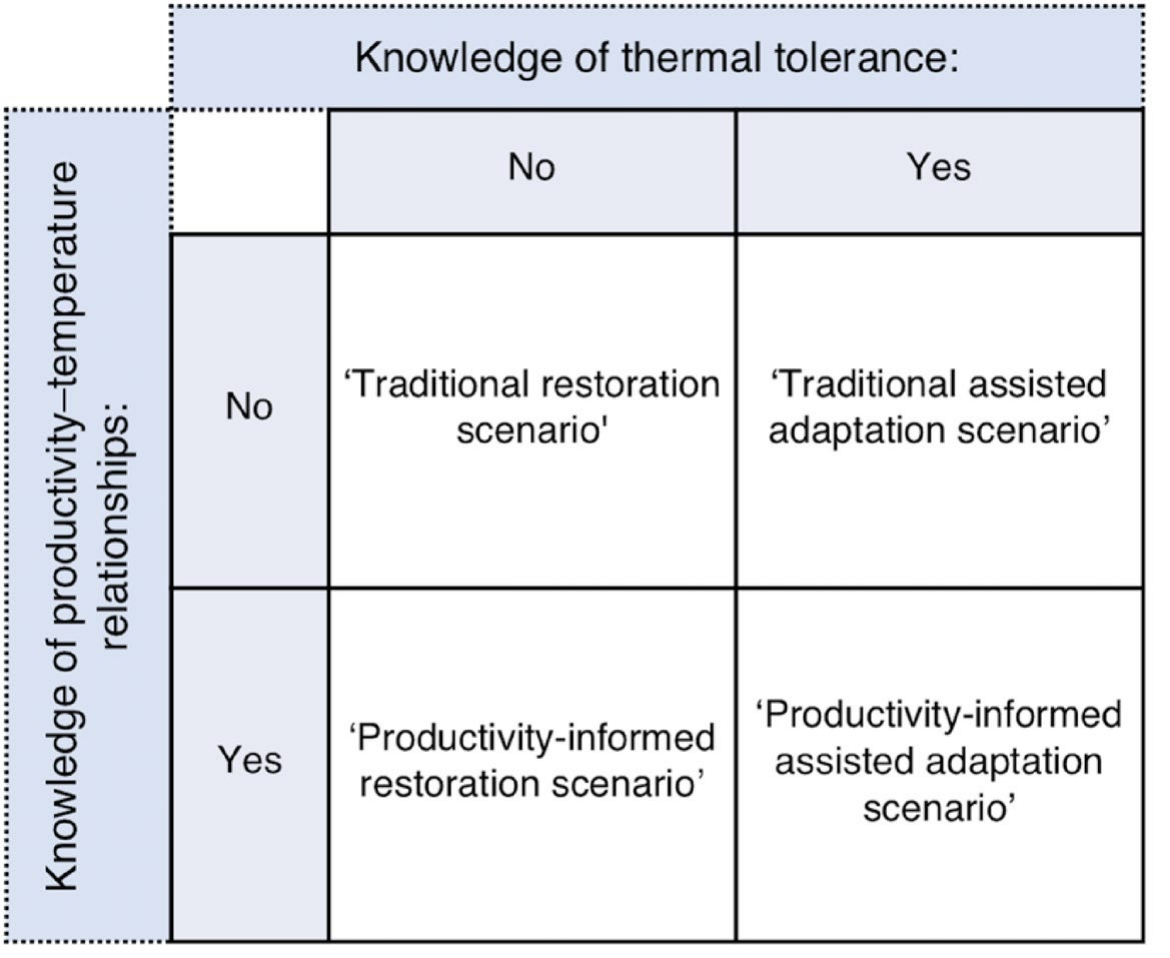
The four restoration and assisted adaptation scenarios assessed and how these are informed by relationships between productivity and temperature and/or knowledge of kelp thermal tolerance.

Here we explore outcomes of restoration and assisted adaptation scenarios on the productivity of kelp used in proactive interventions under future climate change. We then examine whether these outcomes can be improved by utilising prior knowledge of how kelp productivity scales with temperature in the design of restoration and assisted adaptation strategies. Outcomes of restoration and assisted adaptation strategies under future ocean warming are derived from empirical relationships between productivity and temperature in this study given that temperature (1) is a dominant driver of kelp productivity^20,21^, (2) correlates strongly with additional environmental predictors of productivity (e.g. photosynthetically available radiation, nitrate availability and chlorophyll *a* concentration) and, (3) is available in the form of downscaled spatial projections that can support anticipatory assessments of future climate adaptation interventions. We use data from Western Australian kelp forests (*Ecklonia radiata*) sampled across a 500 km latitudinal gradient to quantify temperature–productivity relationships that have a strong genomic basis^22^ and signatures of selection for thermal tolerance^8^. These data facilitate the assessment and comparison of four restoration and assisted adaptation scenarios under climate change (Fig. 1).

The first is a ‘traditional restoration scenario’ that utilises the mean temperature–productivity response to evaluate how ocean warming is likely to affect the productivity of restored kelp if individuals are randomly selected from the population for use in restoration projects. Given this scenario utilises the mean population response, it also reflects how ocean warming is likely to affect kelp productivity in the absence of any climate interventions. The second scenario simulates a ‘productivity-informed restoration scenario’ by utilising the temperature–productivity relationship for the top 10% most productive individuals from each sampling location and period throughout the study region. This scenario is based on the potential for local restoration projects to be informed by temperature–productivity relationships and utilises individuals that are highly productive to improve benefits of restored kelp under climate change. Genetic variation in heat tolerance has recently been experimentally linked to positive outcomes for productivity and other key traits in subsequent life stages in *E. radiata*^23,24^ and other kelps^25^. Within sampling locations, highly productive adult sporophytes result in highly productive gametophytes^26^. Moreover, we have recently confirmed the strong genomic basis for high productivity^22^ demonstrating that productivity can be a heritable trait. Scenario three simulates a ‘traditional assisted adaptation scenario’ by utilising the mean temperature–productivity relationship for individuals from the equatorward range-edge (Jurien Bay, Western Australia). Individuals from this location are associated with heritable genetic traits linked to putative selection for temperature tolerance^8,24^ making them suitable candidates for use in assisted adaptation efforts at other sites. Finally, scenario four simulates a ‘productivity-informed assisted adaptation scenario’ by using the temperature–productivity relationship for the top 10% most productive individuals from the equatorward range-edge (Jurien Bay). This scenario explores whether the productivity of kelp used in assisted adaptation efforts can be enhanced through prior knowledge of productivity–temperature relationships specifically for thermally tolerant individuals.

We use these four scenarios to quantify changes in productivity between present-day kelp (2010-centered period) and kelp used in intervention efforts under mid-century (2050-centered period) ocean warming conditions. Comparisons among scenarios highlight how kelp productivity may be eroded or enhanced under climate change depending on the individuals selected for use in interventions. This work illustrates the need to strategically select individuals from natural populations for use in marine restoration and assisted adaptation programs to ensure the benefits of investment in proactive interventions are optimised under climate change.

## Methods

### Kelp productivity–environmental relationships

Kelp productivity, measured as grams of fresh weight produced per individual per day (g FW ind^− 1^ d^− 1^), was quantified at three locations (Jurien Bay [30.32°S], Marmion [31.80°S] and Hamelin Bay [34.24°S]) extending across 500 km of latitude along the Western Australian coast. The oceanography of these locations is dominated by the poleward following Leeuwin Current, which weakens during the Austral summer when opposing winds prevail and strengthens during winter when opposing winds cease. The benthic habitat in each sampling location consists of a mosaic of patch reefs dominated by *E. radiata* and interspersed with sandy substrate. Kelp productivity was measured at three sites per location that were separated by at least 1 km and consistently located within depths of 8–10 m. This was repeated over three six-week periods during the Austral autumn, spring and summer at all locations over one year (March 2011–February 2012).

Kelp productivity was measured for fifteen randomly selected individuals per site during each six-week period (*n* = 405) by applying the widely utilised ‘hole-punch method’^27,28^. This was done by punching two holes into the central lamina of individuals at the beginning of each six-week monitoring period, with the first hole located 5 cm from the junction between the stipe and lamina and the second hole located 10 cm from this junction. Given that new growth of *E. radiata* tissue occurs in this location and older material translocated towards the distal tips of the lamina, the extension of tissue here is a reliable measure of growth rate. Following each six-week monitoring period, the distance from the first hole to the stipe–lamina junction and the distance between the two holes were recorded for each individual in the laboratory. Thallus extension was then calculated by subtracting the sum of these two measures by 10 cm. To convert rates of thallus extension to kelp productivity, 5 cm wide segments (perpendicular to the central lamina) were sectioned from above the stipe-lamina junction. The segment (5 cm) of maximum biomass (for the first 30 cm) was then used to calculate kelp productivity (*P*; g FW ind^− 1^ d^− 1^) during the monitoring period following:

$$\:P=e\times\:\frac{FW}{5}/t$$

where *e* is the observed thallus extension (cm), *FW* is the fresh weight (g) of the heaviest strip (divided by 5 to give g cm^− 1^ of lamina), and *t* is the number of days between punching holes and collecting kelp^29,30^.

To assess for environmental drivers of kelp productivity throughout the study region, multiple variables known to influence the distribution and growth of macroalgae were compiled for analysis. These were temperature (°C), photosynthetically available radiation (PAR; mol m^− 2^ d^− 1^), nitrate availability (NO3^−^; mmol m^− 3^) and chlorophyll *a* concentration (CHL; mg m^− 3^). Temperature was recorded at 10-minute intervals at each of the three sites within locations using onset HOBO data loggers (Onset Computer Corporation) moored 1 m off the seafloor. Data loggers failed at two sites during the study period, resulting in a total of 375 individuals for which productivity and temperature data were available for analysis. For all sites that productivity and temperature data were available, PAR, NO3^−^ and CHL were extracted for each sampling location and six-week monitoring period from oceanographic products available from the Copernicus Marine Service (https://data.marine.copernicus.eu/products) and the NASA Goddard Space Flight Center, Ocean Biology Processing Group (https://oceancolor.gsfc.nasa.gov). These were the MODIS-Aqua ocean color product for PAR and the Copernicus global ocean biogeochemical multi-year hindcast (product #001_029) for NO3^−^ and CHL. All environmental data were averaged over each 6-week kelp productivity sampling period prior to analysis.

Environmental variables were assessed for collinearity using pair plots and Pearson’s correlation coefficients (*r*) to evaluate the potential for including multiple environmental predictors in kelp productivity statistical models. High levels of collinearity among predictor variables (i.e. greater than or less than approximately 0.5 and -0.5, respectively) negatively affect model performance through the introduction of multicollinearity (violating the assumption of predictor independence)^31^ resulting in unstable coefficient estimates, inflated standard errors and compromised model interpretability and predictive skill^32^. We found temperature to be strongly correlated with PAR (*r* = 0.45; Fig. S1a), NO3^−^ (*r* = -0.58; Fig. S1b) and CHL (*r* = -0.91; Fig. S1c). Only variables with the clearest ecological interpretation, or those for which data are readily available to facilitate model prediction, among covarying pairs are recommended to be retained for statical modelling^32,33^. Furthermore, multifactor manipulative experiments demonstrate that temperature usually plays a much larger role in impacting the physiology of kelp than nutrients across life stages^34,35^. Therefore, temperature was retained for further analyses.

Linear models were initially fitted to assess for relationships between kelp productivity and temperature within the full dataset (i.e. the traditional restoration scenario) and the three subsets of these data that reflect alternative restoration and assisted adaptation scenarios (Fig. 1). Residuals from all linear models fitted were compared to additional factors (i.e. sampling location and kelp total weight) potentially associated with intraclass correlation structures^33^. This process identified a significant positive relationship between kelp productivity and kelp weight (*p* < 0.01; Figs. S2a and S2b), indicating that the modelled relationship between kelp productivity and temperature could be improved if kelp weight (100 g weight bins; categorical variable) was included as a random intercept term in all models. Therefore, the resulting linear mixed effects models trained on the full dataset and subsets representing each of the restoration and assisted adaptation scenarios took the form (in script notation):

$$\:Response=Temp+\left(1\right|Weight)$$

where *Response* is kelp productivity, *Temp* is in situ temperature and *Weight* is kelp weight included as a random effect to account for variation in productivity being driven by kelp size (Fig. S2c). Biomass erosion was also measured in the field for each individual and these data were also regressed against the mean temperature recorded during each monitoring period, but no significant relationship was found (*p* = 0.66, *r*^2^ = -0.002; Fig. S3).

To evaluate and compare the predictive performance of the four models representing each of the restoration and assisted adaptation scenarios, we randomly sampled 20 observations from each training dataset to act as test sets, ensuring comparability among models trained on different data subsets. Predictive performance was quantified using three complementary metrics. Root Mean Square Error (RMSE) and Mean Absolute Error (MAE) were used to assess model prediction accuracy, with lower values indicating better performance. Additionally, model bias was calculated as the mean prediction error to assess for systematic over- or under-prediction tendencies represented by positive and negative values.

### Present-day and mid-century kelp productivity

Spatial predictions of kelp productivity for the period encompassing 2001–2020 were computed throughout the study extent (28–36°S, 110–120°E) using the mean population response (i.e. traditional restoration scenario) and observed (level 4) sea surface temperature (SST) data obtained from the Copernicus Marine Service (0.05° spatial resolution; product #010_011). While satellite-derived SST and in situ temperatures measured by data loggers are highly comparable throughout the study extent^36^, there are likely to be instances where temperature varies with depth throughout the water column. Given that field-derived relationships between kelp productivity and temperature utilised in situ data, we converted SST to sea bottom temperature (SBT) by applying a delta change factor analysis (e.g^37–39^. This was done by calculating the difference (i.e. delta values) between mean annual SST and SBT produced by the GLORYS12V1 physical oceanographic reanalysis model (Copernicus Marine Service product #010_011) over the 2001–2020 period for each grid cell throughout the study extent. Delta values between modelled SST and SBT were then applied to observed mean annual SST data to calculate SBT for the present-day period (i.e. 2001–2020). While SBT data directly from the physical oceanographic reanalysis model may have been suitable for producing spatial predictions of kelp productivity, the delta change factor analysis was applied to observed SST to minimise the influence of model bias on kelp productivity estimates while also accounting for potential variation in temperature with depth.

To produce spatial projections of kelp productivity under mid-century ocean warming, an ensemble of SST data encompassing a 20-year period (2041–2060) centred on 2050 was downscaled from seven global climate models (GCMs; CMIP6; Table S1). GCMs were selected using the IPCC Working Group 1 Interactive Atlas of Regional Information interface (https://interactive-atlas.ipcc.ch/regional-information) to ensure the models used to develop the multi-model ensemble encompassed a range of ocean temperature futures projected under climate change. SST projections under the SSP5-8.5 scenarios were extracted from the CMIP6 data portal for analysis (https://esgf-node.llnl.gov/search/cmip6). This high emissions scenario reflects continued fossil fuel development and was selected for use in this study given that the implementation of restoration and assisted adaptation scenarios will be most urgently required under rapid ocean warming.

The coarse spatial resolution of GCM data (0.25–1°) limits its utility for projecting changes in kelp productivity in coastal environments. Therefore, the delta method (e.g^38,39^. was used to downscale SST data from CMIP6 models following the methods detailed by Champion et al.^40^. Briefly, this process involved (1) calculating the difference between mean annual SST data for the period 2041–2060 and a modelled present-day baseline period encompassing 2001–2020 for each CMIP6 model forced under SSP5-8.5, (2) bilinearly interpolating delta value matrices from their native model resolution (~ 1°) to the finer resolution of observed ocean data (i.e. 0.05°), and (3) adding delta values to mean annual SBT data (derived from observed SST data as described above) that encompassed the period 2001–2020 (where we assume differences between SST and SBT remain consistent for shallow marine environments under climate change). These steps produced mean annual SBT data for the period encompassing 2041–2060 (i.e. the 20-year mean centred on 2050) downscaled to a common 0.05° resolution from seven CMIP6 models forced under SSP5-8.5. A multi-model ensemble was then created using median SBT values from downscaled projections across each of the seven GCMs. Median values were utilised to create the multi-model ensemble as this statistic is robust to outliers and does not assume the data are normally distributed.

Utilising the ensemble of downscaled SBT data, spatial projections of kelp productivity for the 2050-centered future period were generated for all restoration and assisted adaptation scenarios throughout the study extent. Latitudinal means of kelp productivity were calculated for present-day and mid-century kelp productivity scenarios. Finally, *t*-tests were applied to test for differences among scenarios and between present-day and mid-century timepoints. These comparisons were spatially restricted to the region encompassing the most equatorward (i.e. Jurien Bay [30.32°S]) and poleward (i.e. Hamelin Bay [34.24°S]) locations where field measurements were taken to avoid statistically analysing model projections that extrapolated beyond the spatial extent of empirical data. All statistical analyses were undertaken using the R programming language^41^ and spatial projections were compiled and plotted in MATLAB (ver. 9.2, The MathWorks, Inc.).

## Results

**Fig. 2:**
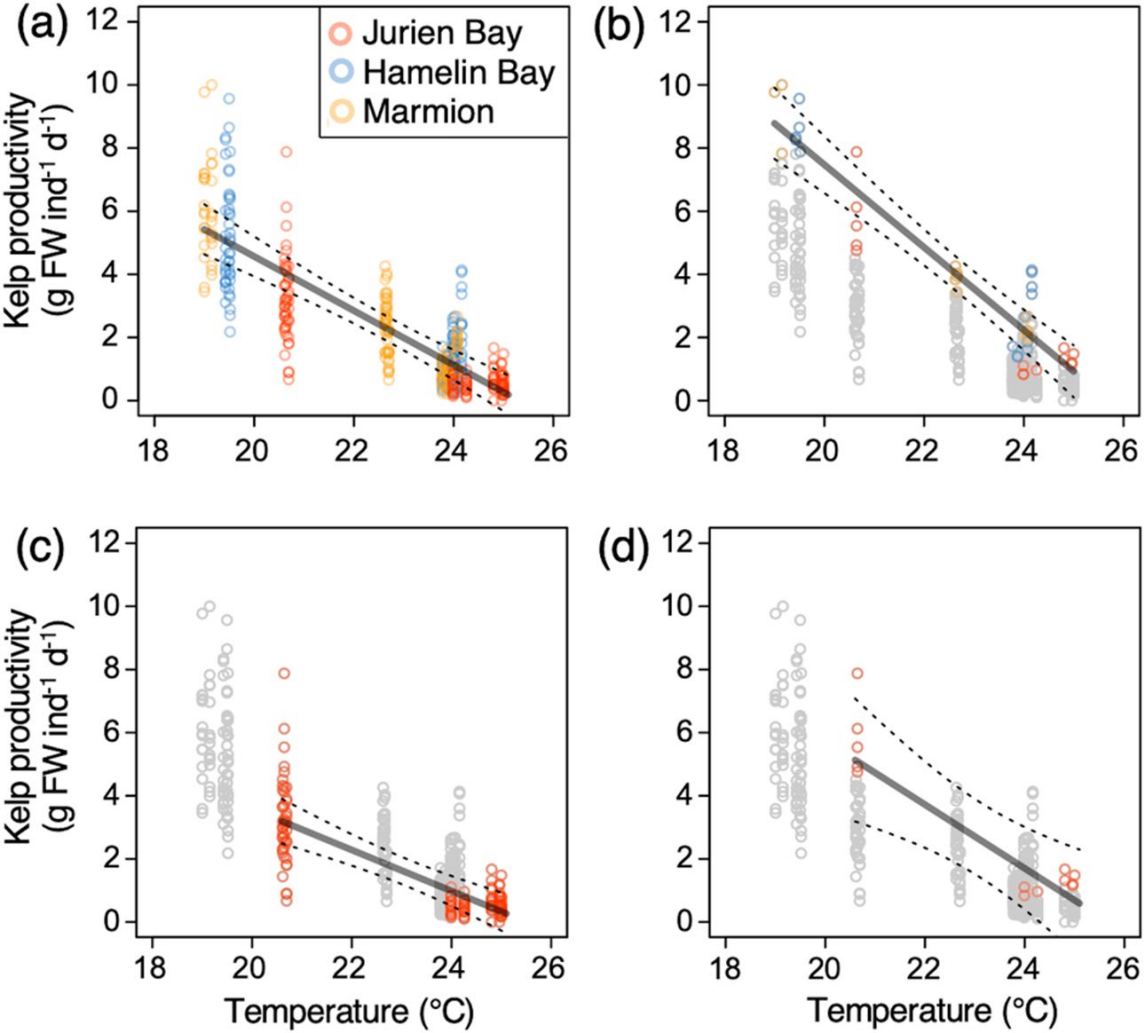
Relationships between kelp productivity (g FW ind^−1^ day^−1^) and mean in situ temperature for *Ecklonia radiata* from Western Australia. Panel (**a**) shows the relationship between productivity and temperature for all individuals (*n* = 375) sampled and reflects kelp productivity likely to be achieved under a traditional restoration scenario where individuals are randomly selected from the population for use in restoration efforts. Panel (**b**) shows the relationship for the top 10% most productive individuals at each sampling location, which represents a productivity-informed restoration scenario where highly productivity individuals are strategically selected for use in restoration efforts. Panel (**c**) shows the relationship for individuals sampled at the species warm-range edge (i.e. Jurien Bay) and represents a traditional assisted adaptation scenario given that heritable genetic traits linked to selection for temperature tolerance are known to occur in these individuals. Panel (**d**) shows the relationship for the top 10% most productive individuals sampled at the warm-range edge, which represents a productivity-informed assisted adaptation scenario where highly productive individuals that also have heritable genetic traits linked to temperature tolerance can be selected for use in assisted adaptation efforts. Dashed lines around model fits represent 95% confidence intervals. Grey data in panels (**b**), (**c**) and (**d**) were excluded when fitting these relationships.

There was a significant negative relationship between kelp productivity (g FW ind^− 1^ d^− 1^) and temperature for Western Australian kelp (*F*_1,373_ = 239.9, *p* < 0.001; Fig. 2). Across the full range sampled, kelp productivity was found to decline by 0.86 g FW ind^− 1^ d^− 1^ for every 1 °C increase in temperature (0.80–0.92 g FW ind^− 1^ d^− 1^ 95% CI; Fig. 2a).

**Fig. 3:**
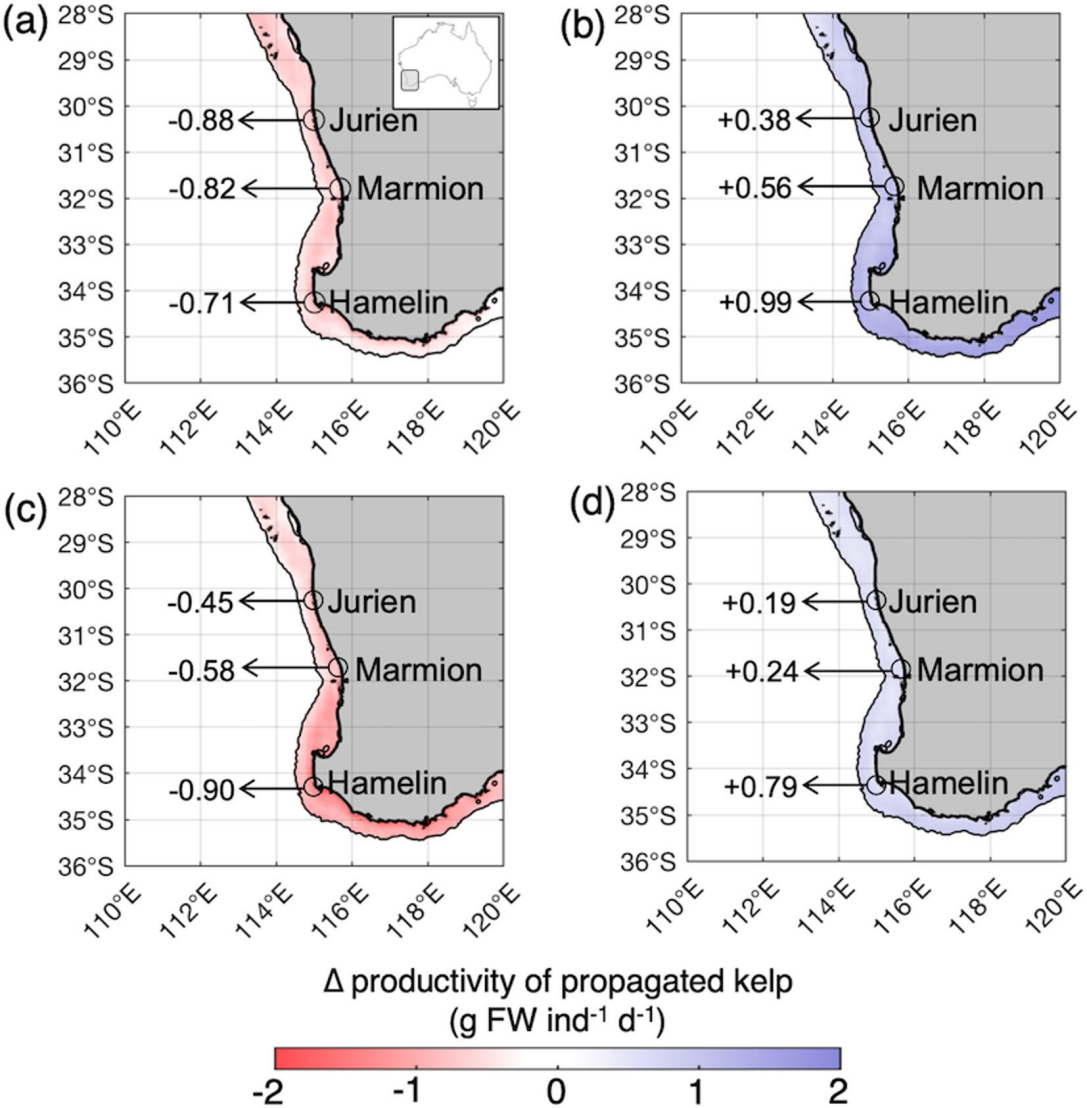
Projected changes to mean annual kelp productivity (g FW ind^−1^ day^−1^) between present-day (2001–2020) and mid-century (2041–2060) periods using (**a**) a traditional restoration scenario (Fig. 2a), (**b**) a productivity-informed restoration scenario (Fig. 2b), (**c**) a traditional assisted adaptation scenario (Fig. 2c) and (**d**) a productivity-informed assisted adaptation scenario (Fig. 2d). Spatial projections do not account for the distribution of suitable hard substrate required for kelp persistence but rather reflect likely changes in productivity when suitable habitat is available.

**Fig. 4:**
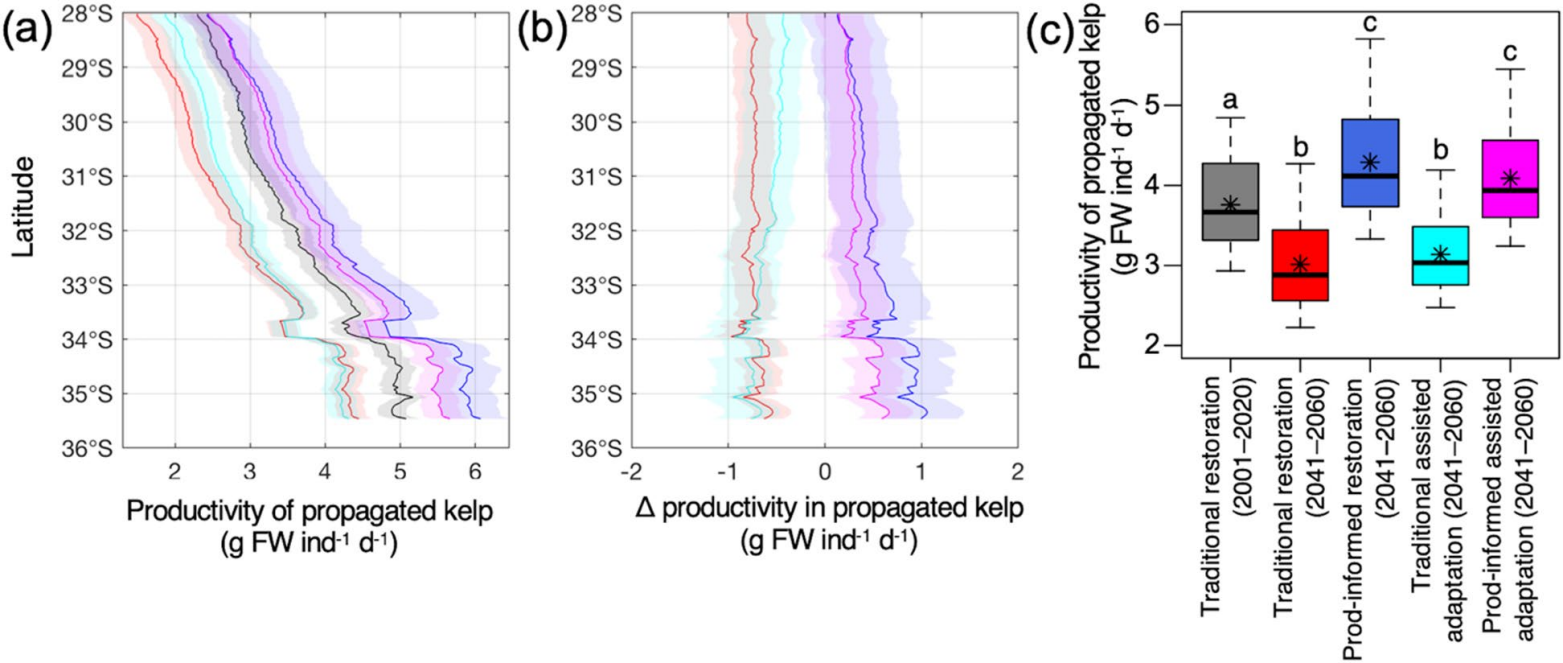
Projected latitudinal trends in (**a**) productivity (g FW ind^− 1^ d^− 1^) and (**b**) changes to productivity by the mid-century throughout the distribution of Western Australian kelp (*Ecklonia radiata*). Grey data denote the full population response (i.e. the traditional restoration scenario; Fig. 2a) projected for the present-day period (2001–2020), red data denote the traditional restoration scenario projected for the mid-century period (2041–2060), dark blue data denote the productivity-informed restoration scenario (Fig. 2b) projected for the mid-century period, cyan data denote the traditional assisted adaptation scenario (Fig. 2c) projected for the mid-century period, and magenta data denote the productivity-informed assisted adaptation scenario (Fig. 2d) projected for the mid-century period. Shaded areas in panels (**a**) and (**b**) represent 95% CI. Boxplots in panel (**c**) depict projected kelp productivity under each intervention scenario assessed throughout the region of Western Australia encompassing the extent of field-derived empirical data analysed herein (i.e. between 30.32 and 34.24° S). Lower-case letters in panel (**c**) indicate the results of significance tests between the means of each group, where data sharing a letter are not significantly different (at alpha level 0.05).

Future projections revealed that kelp productivity could decline by up to 22% (20.7–22.3% 95% CI; ~0.75 g FW ind^− 1^ d^− 1^) throughout Western Australia under mid-century ocean warming (Figs. 3a and 4). This result is the product of undertaking future projections using the productivity–temperature response from the entire range sampled (Fig. 2a), which is analogous to a traditional restoration scenario whereby individuals are randomly sampled throughout each population for use in restoration efforts. Therefore, the productivity of kelp used in a traditional restoration context under mid-century conditions is projected to be significantly lower than average present-day kelp productivity (Figs. 3a and 4; *t*_218_ = 10.15, *p* < 0.001). Similarly, the productivity of kelp restored using the traditional assisted adaptation scenario (Fig. 2c) is projected to decline by 16% (16.1–16.5% 95% CI; ~0.60 g FW ind^− 1^ d^− 1^) throughout Western Australia under mid-century ocean warming relative to the present-day (Figs. 3c and 4). These rates of productivity were also significantly lower than those found for the present-day period (Fig. 4c; *t*_218_ = 9.18, *p* < 0.001). There was no significant difference in rates of productivity projected using traditional restoration and traditional assisted adaptation scenarios under mid-century ocean warming (Fig. 4; *t*_218_ = -1.83, *p* = 0.07).

In contrast, the productivity-informed restoration scenario (Fig. 2b) could potentially increase the productivity of restored kelp by up to 14% (13.2–14.1% 95% CI; ~0.52 g FW ind^− 1^ d^− 1^) under mid-century ocean warming relative to average present-day kelp productivity (Figs. 3b and 4). Rates of mid-century kelp productivity projected using the productivity-informed restoration scenario were significantly greater than those using traditional restoration and traditional assisted adaptation scenarios (Fig. 4c). Furthermore, rates of productivity associated with the productivity-informed restoration scenario were significantly greater under mid-century conditions than average present-day kelp productivity (Fig. 4; *t*_218_ = -6.48, *p* < 0.001).

Application of the productivity-informed assisted adaptation scenario (Fig. 2d) was also found to enhance the productivity of restored kelp by approximately 9% (9.0–9.4% 95% CI; ~0.34 g FW ind^− 1^ d^− 1^) under mid-century conditions relative to average kelp productivity projected for the present-day period (Figs. 3d and 4). Rates of kelp productivity projected for the mid-century using the productivity-informed assisted adaptation scenario were significantly greater than those projected using the traditional restoration and traditional assisted adaptation scenarios (Fig. 4). However, mid-century kelp productivity projected using the productivity-informed restoration scenario marginally exceed rates of kelp productivity projected using the productivity-informed assisted adaptation scenario (Fig. 4), however these differences were not statistically significant.

Our assessment of the predictive performance of each model used to project future kelp productivity under each restoration and assisted adaptation scenario revealed that all models were associated with reasonable and comparable predictive capacity (Table S2). For example, RMSE and MAE values for all models ranged between 0.870 and 1.063 and 0.636–0.779, respectively. All models exhibited minimal systematic bias (range: -0.099–0.256), indicating well-calibrated predictions without substantial over- or under-estimation tendencies.

## Discussion

Understanding how the productivity of marine habitats varies in response to environmental conditions presents an underexplored opportunity for strategically maximising the benefits of climate interventions. The potential for this opportunity is supported by experiments and genomic data demonstrating that any population can contain genotypes that are exceptional performers in terms of productivity^22,25,26^ and survival under thermal stress^6^. The importance of exploring scenarios that have the potential to enhance the productivity of individuals used in restoration and assisted adaptation interventions is underscored by the ~ 22% decline to productivity projected for Western Australian kelp by the mid-century in response to ocean warming.

Analysis of the scenarios investigated here demonstrate that strategically selecting highly productive individuals across a range of temperatures for use in restoration and assisted adaptation efforts can achieve rates of productivity under mid-century ocean warming that exceed the present-day average. Given the genetic heritability of productivity in these populations^22^ and the genomic underpinnings of temperature tolerance^8^, these traits can be passed on to future generations, ensuring that benefits to these values are enduring. Specifically, application of the productivity-informed restoration scenario resulted in restored kelp that were ~ 14% more productive throughout Western Australia under mid-century conditions than average levels of present-day kelp productivity. These projections are based on the strategic selection of the top 10% most productive individuals for use in restoration efforts, measured across a latitudinal gradient characterised by ~ 6 °C of thermal variation. While we have empirical evidence for *E. radiata* that productivity within sites has a strong genomic basis and is likely heritable^22^ and that this relationship is stable across temperatures^26^, productivity remains a highly variable trait that can be influenced by multiple environmental factors. For this reason, results from our productivity-informed restoration scenario reflects likely levels of productivity associated with the first generation of individuals utilised in restoration efforts (i.e. donor adult individuals or gametophytes). Experimental transplants could unravel how successive generations will perform, especially as selected individuals outcross with local genotypes.

Assisted adaptation strategies can be incorporated into restoration and are designed around the principle of selecting and propagating known genotypes that have been shown to be better adapted to future conditions, such as higher temperatures^42^. These individuals are often identified through genomic studies demonstrating heritable loci under selection linked to higher temperatures^9^, which for kelps^8^ and many other organisms^43^ are found in greater frequency in warm, low latitude populations. For kelp off Western Australia, these genotypes occur in higher frequency from Jurien Bay northwards^8^. Moreover, the frequency of these equatorward genotypes has naturally increased in poleward populations following extreme thermal events, presumably because they are fitter and survive under thermal stress^42^. Individuals from locations such as Jurien Bay would, therefore, be logical to use in restoration strategies that include an element of assisted adaptation to boost heritable thermal tolerance elsewhere throughout the distribution of Western Australian kelp^11^. Thus, we assessed the outcome of assisted adaptation strategies on kelp productivity using the relationship between productivity and temperature for individuals from Jurien Bay only. These assisted adaptation scenarios were modelled with (productivity-informed assisted adaptation scenario) and without (traditional assisted adaptation scenario) prior knowledge of how productivity scales with temperature among individuals at Jurien Bay. Contrary to expectations, the productivity of kelp used in the traditional assisted adaptation scenario was ~ 16% lower throughout Western Australia under mid-century projections relative to average kelp productivity projected for the present-day period. This decline is comparable to results produced using the traditional restoration scenario (~ 14% decline projected relative to present-day productivity). However, given that individuals from the equatorward range-edge at Jurien Bay are associated with genotypic characteristics that make them thermal resilient^6^, randomly selecting individuals from this location for use in intervention efforts is still likely to contribute blue carbon, habitat provision and ecosystem benefits in ways other than through productivity. For example, through long-term persistence and survival of short-term extreme events such as marine heatwaves.

Projections using the productivity-informed assisted adaptation scenario revealed clear benefits of using highly productive individuals from the thermally resilient populations in restoration efforts. This scenario was associated with a ~ 9% increase in productivity under mid-century ocean warming throughout Western Australia relative to the present-day. Under mid-century ocean warming, these levels of productivity were markedly higher than those projected using both the traditional restoration and assisted adaptation scenarios and only marginally lower (up to 5% difference in average productivity) relative to the productivity-informed restoration scenario. The apparent capacity for the productivity-informed assisted adaptation scenario to enhance the productivity of restored kelp under ocean warming above present-day levels, while simultaneously harnessing the benefits of using genotypes associated with heritable thermal resilience, makes this scenario particularly advantageous. As for all scenarios assessed, the productivity of kelp restored using the productivity-informed assisted adaptation scenario displayed a latitudinal trend, with greater productivity associated with kelp restored in higher latitudes. Taken together, our projections suggest that the productivity-informed assisted adaptation scenario applied in relatively high latitude locations is likely to yield the greatest benefits when undertaking kelp forest restoration.

Environmental factors others than temperature contribute to the growth and productivity of macroalgae^20^, including *E. radiata*^21,23^. Our analyses of additional factors revealed strong collinearity between temperature and photosynthetically available radiation, nitrate availability and chlorophyll *a* concentration over the study period and region (Fig. S1). Given the presence of these correlation structures, modelling kelp productivity as a function of temperature alone implicitly included the influence of additional environmental factors while retaining the clear influence of temperature on the productivity response (Fig. 2). This approach also facilitated the assessment of kelp productivity within restoration and assisted adaptation scenarios under future ocean warming as downscaled spatial projections for a mid-century time point were only available for temperature. However, it is important to recognise that our analyses assume that existing correlation structures between temperature and photosynthetically available radiation, nitrate availability and chlorophyll *a* concentration will persist into the future. Furthermore, projections of kelp productivity were produced by temperature–productive models that took the same form but were trained on unique subsets of data representing each restoration and assisted adaptation scenario. Despite this, predictive performance was found to be highly comparable among models and free of systematic over- or under-prediction (Table S2). These findings do not preclude the influence of model uncertainty on the precision of our results but rather suggest that their directional effects towards higher future kelp productivity under the productivity-informed restoration and assisted adaptation scenarios are robust to model uncertainty.

While our analyses provide a robust comparison of future outcomes for kelp productivity under multiple restoration and assisted adaptation scenarios, how these translate to blue carbon, habitat provision and broader ecosystems benefits is contingent on additional factors that were beyond the scope of modelling in this study. For example, the transport and fate of restored kelp populations is of crucial importance to its blue carbon potential. Kelp forests account for approximately 30% of the total blue carbon stored around the Australian continent^15^ and dense shelf water transport has been shown to effectively transport up to 51% of kelp detritus to the deep ocean within the study region assessed here^17^. While these results demonstrate mechanisms linking the productivity of restored kelp and its blue carbon sequestration potential in the deep ocean under present-day conditions, transport mechanisms and their connections to restoration sites may vary due to climate-driven changes in ocean circulation. However, there is also potential for off-shelf transport of kelp detritus to increase in the future, subsequently enhancing the contribution of kelp forests to blue carbon storage and sequestration. Climate-driven changes to biotic interactions may also impact the productivity values associated with restored kelp forests. For example, shifts in the distribution of kelp grazers^44,45^ may mediate the benefits derived from restoring highly productive kelp in the future. In some regions, kelp may experience increased herbivory that reduce habitat provision values, while declines in herbivory in other regions may manifest in enhanced blue carbon and ecosystem benefits. Addressing these questions presents a valuable avenue for future research that would require embedding the scenarios evaluated here within coupled ecosystem and oceanographic models. Nevertheless, our projections illustrate how alternative approaches for sourcing individuals for use in restoration effects have the potential to alter benefits associated with restored kelp under future warming.

Climate-driven environmental change presents numerous challenges for the sustainability of kelp forests globally^4,23^, including their productivity which is intrinsically linked to the blue carbon, habitat provision and ecosystem service values of these habitats^5,15^. Our findings demonstrate that restoration and assisted adaptation strategies that are informed by the relationship between kelp productivity and temperature hold potential for enhancing these values for kelp used in interventions under ocean warming. The implementation of productivity-informed interventions would require research to identify individuals associated with relatively high productivity in response to temperature prior to the commencement of on-ground work, followed by biobanking or culturing. However, without the strategic selection of highly productive individuals for use in future kelp interventions, the productivity of restored kelp is likely to approximate the declines projected for the overarching population (here up to a 22% decline in productivity) thereby continuing to undermine the values associated with kelp forest habitats. We advocate for this type of underpinning research to become a principle and future direction of frameworks guiding the restoration of marine habitats under changing environmental conditions.

## Supplementary Information

Below is the link to the electronic supplementary material.

Supplementary Material 1

## Data Availability

Spatial environmental data analysed in this study are available from the Copernicus Marine Service (https:/data.marine.copernicus.eu/products) and the NASA Goddard Space Flight Center, Ocean Biology Processing Group (https:/oceancolor.gsfc.nasa.gov). Kelp productivity data analysed within this study are available from the authors upon reasonable request.
