# Supplementary material for: Enhancing kelp productivity in restoration and assisted adaptation interventions under ocean warming
Source: Sci Rep. 2025 Nov 5;15:38796. doi: 10.1038/s41598-025-22691-y (PMC12589435; doi:10.1038/s41598-025-22691-y)
Supplement: Supplementary file 1 — Supplementary Material 1 [file 41598_2025_22691_MOESM1_ESM.docx]

***Supplementary Material***

**Enhancing kelp productivity in restoration and assisted adaptation interventions under ocean warming**

Curtis Champion^1,2^, Thomas Wernberg^3,4^, Melinda A. Coleman^1,2,3^

^1^NSW Department of Primary Industries and Regional Development, Coffs Harbour, NSW, Australia

^2^National Marine Science Centre, Southern Cross University, Coffs Harbour, NSW, Australia

^3^UWA Oceans Institute and School of Biological Sciences, University of Western Australia, Perth, WA, Australia

^4^ Norwegian Institute of Marine Research, Nye Flødevigveien 20, 4817, His, Norway


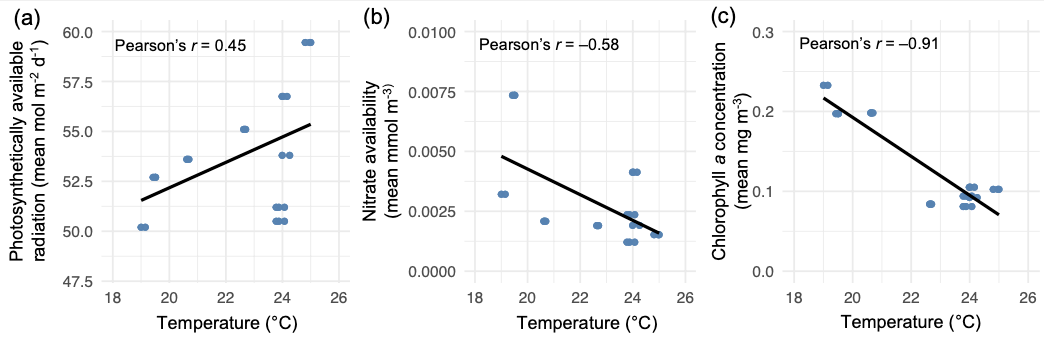


**Figure S1.** Collinearity between in situ temperature measurements and (a) photosynthetically available radiation, (b) nitrate availability and (c) chlorophyll *a* concentration at each sampling site during the study period. Pearson’s correlation coefficients denote substantial collinearity between in situ temperature measurements and each of the three additional environmental variables considered as candidate predictors of kelp productivity.


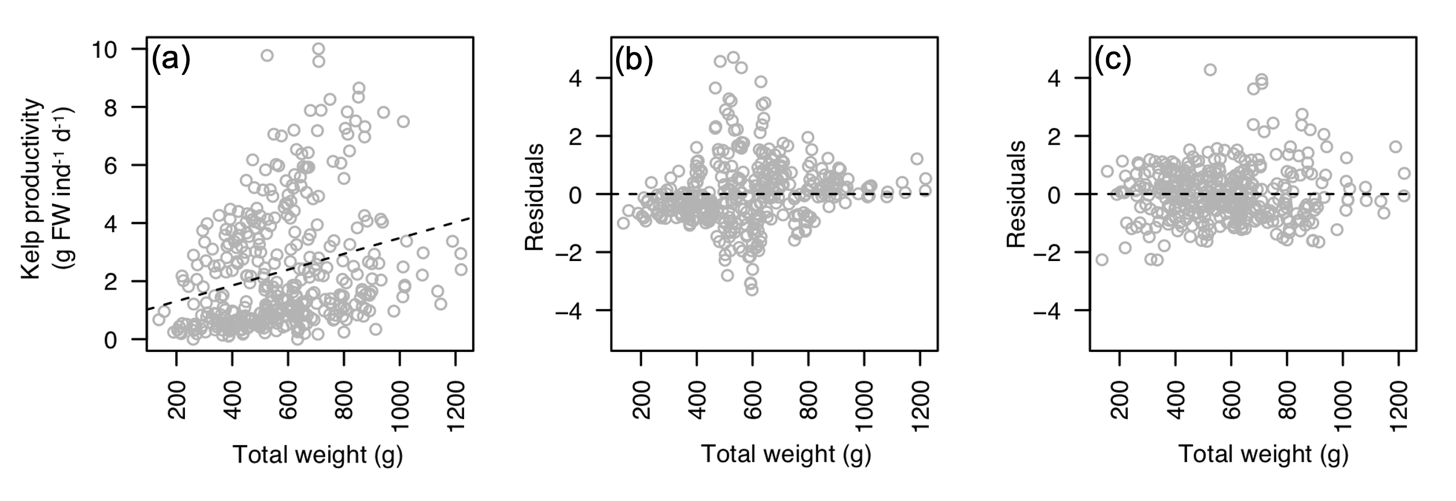


**Figure S2.** Panel (A) Evidence for a significant positive effect of plant weight on biomass production in *Ecklonia radiata* from Western Australia (*F*_1,373_ = 34.62, *p* < 0.001, *r*^2^ = 0.08). Panel (B) Distribution of residuals from a linear model fitted to mean *in situ* ocean temperature and biomass production in *E. radiata* from Western Australia plotted against the total plant weight (100 g weight class bins utilised) displaying a positive relationship between residuals and plant weight highlighting that this model does not fit all data similarly and suggests the need for a mixed effects modelling approach. Panel (C) Distribution of residuals from the final linear mixed effects model (include total weight as a random intercept term) plotted against total plant weight (100 g weight class bins utilised) demonstrating an approximately even distribution of residuals around zero, which indicates the model fitted all data similarly.


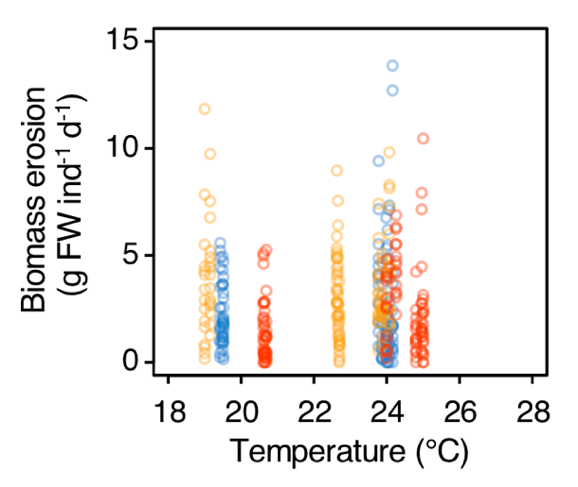


**Figure S3.** Data demonstrating no relationship between mean *in situ* temperature and kelp biomass erosion for *Ecklonia radiata* from Western Australia (*p* = 0.66, *r*^2^ = –0.002). Red, blue and orange data denote individuals measured at Jurien Bay, Hamelin Bay and Marmion locations, respectively.

**Table S1.** Summary of global climate models downscaled (0.05°) to support future projections of kelp productivity within intervention scenarios.

| Model | Institution | Native resolution (°) |
| --- | --- | --- |
| ACCESS-CM2 | Commonwealth Scientific and Industrial Research Organisation (CSIRO) | 1.0×1.0 |
| ACCESS-ESM1-5 | Commonwealth Scientific and Industrial Research Organisation (CSIRO) | 1.0×0.0 |
| GFDL-CM4 | National Oceanic and Atmospheric Administration (NOAA) | 0.25x0.25 |
| GFDL-ESM4 | National Oceanic and Atmospheric Administration (NOAA) | 0.5×0.5 |
| MIROC6 | Japan Agency for Marine-Earth Science and Technology (MIROC) | 1.0×1.0 |
| NorESM2-LM | Norwegian Earth System Model Climate Modelling Consortium (NCC) | 1.0x1.0 |
| HadGEM3-GC31-LL | Met Office Hadley Centre (MOHC) | 1.0x1.0 |

**Table S2.** Summary of the predictive performance of each model fitted to assess the outcomes of four restoration and assisted adaptation scenarios on kelp productivity under mid-century ocean warming. Lower Root Mean Square Error (RMSE) and Mean Absolute Error (MAE) values are indicative of better model performance (note that the range of values among models is small). Bias is the mean prediction error, with over- or under-prediction tendencies represented by positive and negative values (note the absence of systematic over- or under-prediction).

| Model | Measure of predictive performance | | |
| --- | --- | --- | --- |
|  | RMSE | MAE | Bias |
| Traditional restoration scenario | 1.063 | 0.727 | -0.099 |
| Productivity-informed restoration scenario | 0.986 | 0.779 | 0.256 |
| Traditional assisted adaptation scenario | 0.870 | 0.636 | 0.110 |
| Productivity-informed assisted adaptation scenario | 0.938 | 0.676 | 0.153 |
